# Application and comparison of point-of-care devices for field evaluation of underlying health status of Guatemalan sugarcane workers

**DOI:** 10.1371/journal.pgph.0003380

**Published:** 2024-07-23

**Authors:** Lyndsay Krisher, Diana Jaramillo, Amy Dye-Robinson, Miranda Dally, Jaime Butler-Dawson, Stephen Brindley, Daniel Pilloni, Alex Cruz, Karely Villarreal Hernandez, Joshua Schaeffer, John L. Adgate, Lee S. Newman

**Affiliations:** 1 Center for Health, Work & Environment, Colorado School of Public Health, University of Colorado Anschutz Medical Campus, Aurora, Colorado, United States of America; 2 Department of Environmental & Occupational Health, Colorado School of Public Health, University of Colorado Anschutz Medical Campus, Aurora, Colorado, United States of America; 3 Grupo Pantaleon, Guatemala City, Guatemala; 4 Department of Environmental and Radiological Health Sciences, Colorado State University, Fort Collins, Colorado, United States of America; 5 Division of Pulmonary Sciences and Critical Care Medicine, Department of Medicine, School of Medicine, University of Colorado Anschutz Medical Campus, Aurora, Colorado, United States of America; 6 Department of Epidemiology, Colorado School of Public Health, University of Colorado Anschutz Medical Campus, Aurora, Colorado, United States of America; UMass Chan Medical School - Baystate Medical Center, UNITED STATES OF AMERICA

## Abstract

With chronic disease prevalence on the rise globally, surveillance and monitoring are critical to improving health outcomes. Point-of-care (POC) testing can facilitate epidemiological research and enhance surveillance systems in limited resource settings, but previous research has identified bias between POC devices and laboratory testing. We compared the performance of two POC blood analyzers, the iSTAT handheld (Abbott, Princeton, NJ, USA) and the StatSensor Creatinine (Nova Biomedical, Waltham, MA, USA) to concurrent blood samples analyzed at a local laboratory that were collected from 89 agricultural workers in Guatemala. We measured creatinine and other measures of underlying health status with the POC and the lab blood samples. Pearson correlation coefficients, Bland-Altman plots, no intercept linear regression models and two-sample t-tests were used to evaluate the agreement between the POC and lab values collected across three study days and to assess differences by study day in a field setting. On average there was no observed difference between the iSTAT and lab creatinine measurements (*p* = 0.91), regardless of study day. Using lab creatinine as the gold standard, iSTAT creatinine results were more accurate compared to the Statsensor, which showed some bias, especially at higher values. The iSTAT had good agreement with the lab for sodium and blood urea nitrogen (BUN), but showed differences for potassium, anion gap, bicarbonate (TCO2), glucose, and hematocrit. In this tropical field setting, the research team devised a protocol to prevent the devices from overheating. In limited resource settings, POC devices carry advantages compared to traditional lab analyses, providing timely results to patients, researchers, and healthcare systems to better evaluate chronic health conditions. Technical challenges due to use of POC devices in high heat and humidity environments can be addressed using a standard protocol for transporting and operating the devices.

## Introduction

Chronic disease prevalence is increasing rapidly throughout low and middle-income countries (LMIC) [1, 2]. Surveillance infrastructure for evaluating and monitoring chronic disease in these regions is often limited, in part due to challenges in accessing laboratory testing to facilitate clinical diagnosis and referral [3]. Hand-held, point-of-care(POC) whole blood analyzers have been used widely in clinical practice, especially in high-resource settings that require urgent clinical information to evaluate acute and chronic health conditions, such as in hospital emergency departments and intensive care units [4, 5]. Due to their portability they also have proven useful in remote environment situations [6, 7]. Increasingly, POC devices are being utilized in low-resource settings in LMIC [8, 9] where access to high-quality laboratory testing is frequently limited by factors such as scarcity of trained phlebotomists and technicians, transportation difficulties, lack of refrigeration, delays in sample separation that could impact results, as well as cost. Importantly for patients, the devices typically require a smaller quantity of blood than a standard venous blood draw, sometimes from only a finger stick, and provide fast results in minutes that allow for timely clinical feedback and referral. Thus, they are increasingly being used for medical surveillance in community and workplace settings, and for the same reasons are also used frequently in field research [10, 11].

Previous studies comparing results of POC versus laboratory have generally shown good agreement [12–14]. However, in some cases, results are mixed, and few studies have compared results of POC devices with laboratory venipuncture “gold standard” values in low resource and remote field settings where environmental and other factors could potentially influence results. For example, in previous field research in Guatemala we found a statistical correction was needed for the accurate measurement of POC creatinine from capillary blood in a population of sugarcane workers [15]. A large kidney health surveillance study conducted in South Africa [16] measured POC creatinine using the iSTAT and StatSensor in both capillary and venous blood, as well as laboratory creatinine measured through the commonly used compensated kinetic Jaffe method in the laboratory to calculate estimated glomerular filtration rate (eGFR). Results were compared to iohexol-measured glomerular filtration rate (mGFR). Both the laboratory and the POC devices were found to overestimate mGFR, although the POC devices showed less positive bias compared to the laboratory Jaffe method. Finally, a more recent study carried out in Guatemala and Nicaragua compared the iSTAT and Statsensor devices with lab serum creatinine measures [17] and assessed the accuracy, cost, and practical application of the two POC creatinine devices in agricultural communities and work settings. In terms of the creatinine results, the iSTAT showed the best agreement with the venipuncture values, while the Statsensor exhibited some proportional bias at higher levels of creatinine. The authors concluded that the selection of the appropriate device would depend on the reason for use, including cost and other contextual factors.

Due to the previous published POC observations mentioned above, we used two different POC devices in conjunction with standard laboratory analysis for this study in Guatemala. The goal was to compare side-by-side blood creatinine and other biomarkers of underlying health (e.g., electrolytes, hemoglobin, hematocrit, glucose) measured by the POC devices with the same test results obtained from a local laboratory contracted for the study. In this report we address the following questions: 1. What is the agreement between lab reported and POC device values in the field setting? 2. What is the agreement of reported values of creatinine between the POC devices? 3. Did agreement differ based on study day? We hypothesized that there would be bias in the results of the POC devices compared to the results from the laboratory. This research was conducted in a tropical field setting where temperatures can exceed the operational temperature requirements of the POC devices, and various approaches have been described in the literature of ways to overcome such challenges [18]. Therefore, we further hypothesized that there would be differences in agreement based on study day due to daily differences in temperature. Finally, we report makeshift means to maintain the POC system within manufacturers’ temperature claims in this setting.

## Methods

This sub-study was conducted as part of baseline data collection for a larger research protocol (the “CKDu, Heat and Air Pollution I (CHAP I)” study) and was based on a convenience sample. Participants were at least 18 years of age and employed at the Ingenio Pantaleon in Escuintla, Guatemala as manual sugarcane cutters during the 2021–2022 harvest season. Worker participants provided informed consent and were enrolled in the study in November 2021, just prior to starting work for the season. Per company policy, manual sugarcane cutters passed a pre-employment medical exam prior to hiring, to ensure they were free of any underlying medical concerns that would impact their safety and ability to work. Since the company only hires men for manual sugarcane cutting, all participants in this cross-sectional study were men.

The hand-held iSTAT (Abbott, Princeton, NJ USA) and StatSensor (StatSensor Creatinine, Nova Biomedical, Waltham, MA, USA) devices were used with venous whole blood (iSTAT) and capillary blood samples (StatSensor). Samples were collected concurrently from participants in the morning, inside an open-air gymnasium at the sugarcane mill on one of three consecutive days, referred to hereafter as Day 1, Day 2, and Day 3. Two iSTAT meters and one Statsensor were used for the study. Samples were collected by trained research personnel according to the manufacturers’ manual for both devices. For the iSTAT the CHEM8+ cartridge was used, which measures hemoglobin, hematocrit, sodium, potassium, chloride, total CO2/bicarbonate (TCO2), anion gap, ionized calcium (iCa), glucose, blood urea nitrogen (BUN), and creatinine (enzymatic/amperometric method). The StatSensor measured creatinine (enzymatic/amperometric method) using creatinine test strips from a blood finger stick. Laboratory creatinine was measured in serum prior to freezing using the kinetic Jaffe method (Architect CI4100, Abbott Core Laboratory, Abbott Park, IL, USA). Phlebotomists from the licensed, independent Herrera Llerandi laboratory in Guatemala City performed venipuncture and transported the samples on ice to the laboratory, where technicians performed analyses the same day. Other lab measures were also measured in serum and included those listed above in the CHEM8+ cartridge for comparison to those same results from the iSTAT and were analyzed using standard procedures [19]. Quality control and linearity testing were conducted prior to data collection for the POC devices according to the manufacturer’s instructions, and the Herrera Llerandi laboratory conducts internal controls daily, as well as a monthly external control [15]. The reference ranges set by the laboratory and published by each POC device manufacturer are included in the S1 Table. The reference ranges are very similar or identical between the lab and the POC devices for each of the examined analytes.

Heat exposure was assessed at the study site each day using a wet bulb globe temperature (WBGT) meter (Kestrel 5400, Kestrel Instruments, Boothwyn, PA). WBGT is a composite measure including temperature, humidity, wind speed and solar radiation [20].

### Ethics statement

Written informed consent was obtained from participants and the study was approved by the Colorado Multiple Institutional Review Board (COMIRB #20–0509) in the United States and ZUGUEME Comité de Ética Independiente in Guatemala.

### Statistical analysis

The agreement between the lab and POC measures was assessed visually using Bland-Altman plots [21]. A two-sample t-test was performed to assess differences between POC and lab measurements. The creatinine data were stratified by day of collection and Pearson correlation coefficients were calculated to assess the agreement by day. One-way ANOVA was used to compare the daily observed WBGT. Additionally, sample-specific correction factors were calculated using linear regression models without intercept, and stratified associations were visualized using scatter plots with linear trend lines. To examine the clinical importance of any statistically significant differences observed between the lab and POC results, we assessed the frequency of values that would be considered outside the normal reference range for each measure. Statistical analyses were performed using R version 4.3.1 [22].

## Results

### Study population and climate information

A total of 89 study participants were included in this analysis. The median age was 30 years (range: 18–57). The observed average WBGT at the study site was significantly different across the three days of the study period (*p* <0.001). The average WBGT on Day 1 was 25.8 degrees Celsius (°C) (SD: 0.9) and the maximum was 27.0°C. On Day 2, the average WBGT was 24.9°C (SD: 1.0) with a maximum of 26.3°C, and on Day 3 the average was 25.5°C (SD: 0.5) with a max of 26.3°C.

### Comparison of POC devices and lab creatinine measurements

**Fig 1:**
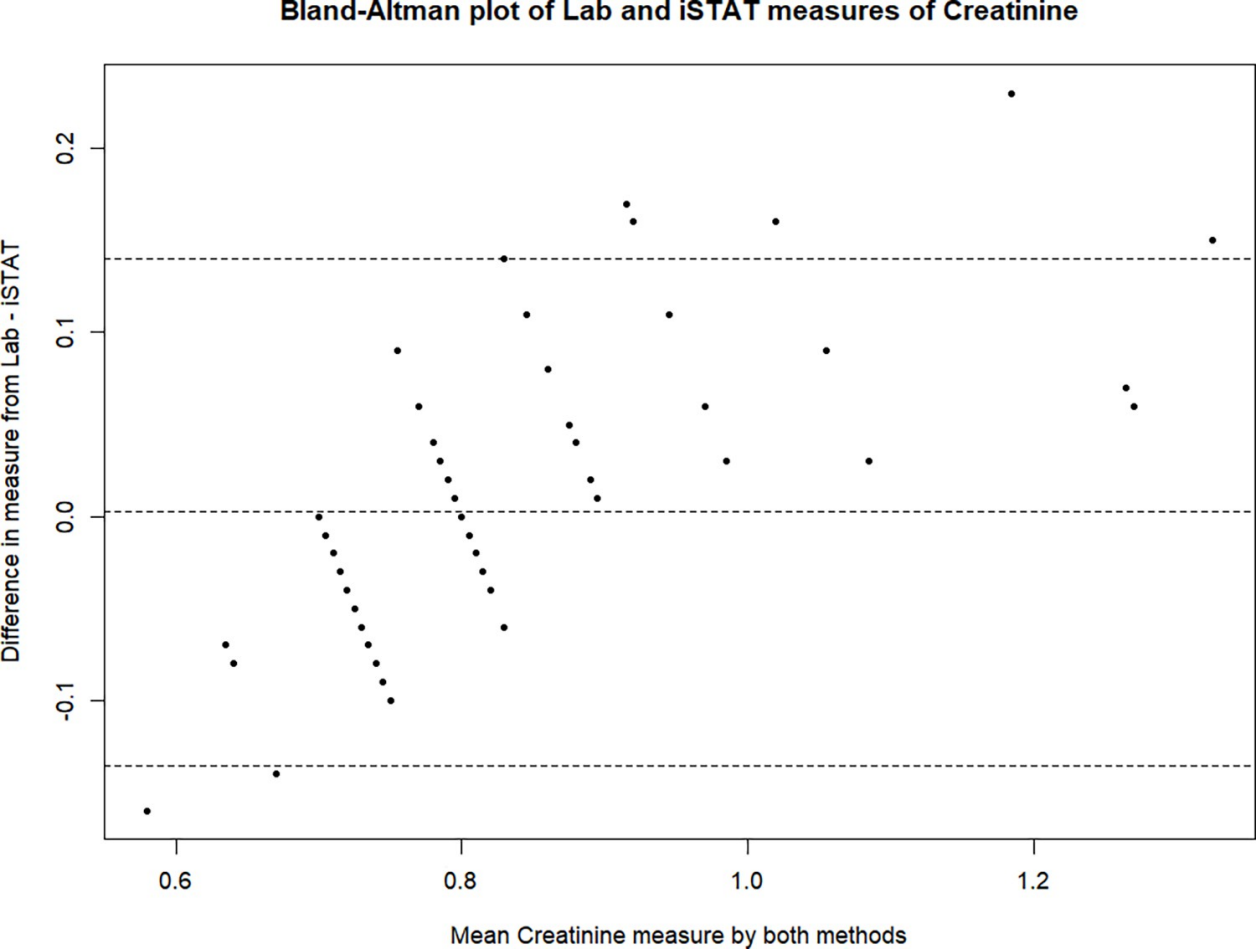
Bland-Altman plot of lab and iSTAT measures of creatinine.

**Fig 2:**
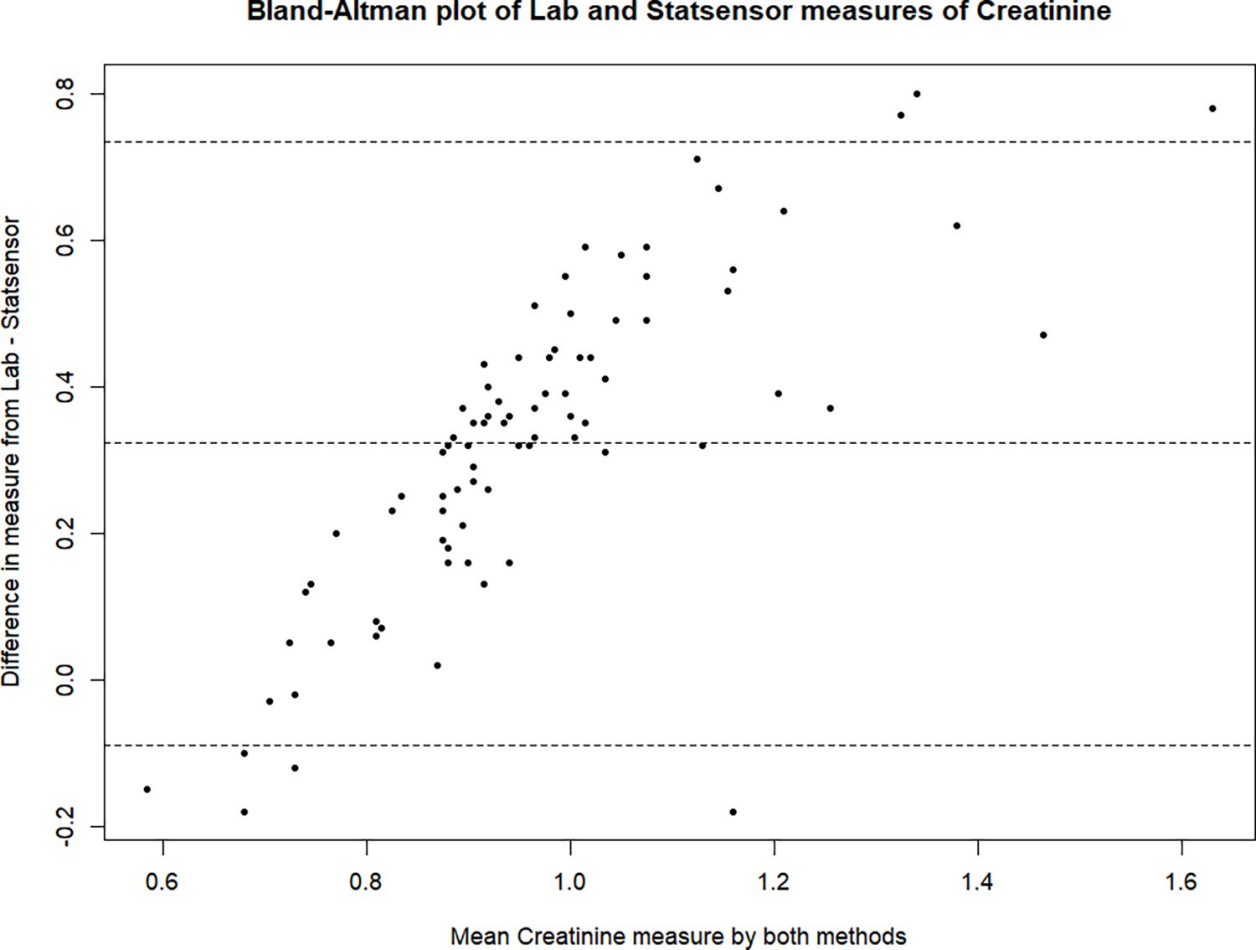
Bland-Altman plot of lab and Statsensor measures of creatinine.

On average there was no observed difference between the iSTAT and lab creatinine measurements (in mg/dL; *p* = 0.91). Shown in Table 1, the mean lab value was 0.80 mg/dL (SD: 0.11) and the mean iSTAT value was also 0.80 mg/dL (SD: 0.16). The mean Statsensor value was 1.12 mg/dL (SD: 0.26) which on average was 0.32 mg/dL (*p* <0.001) higher compared to the lab measurement. The overall pooled adjustment for iSTAT and lab was 0.98. Meanwhile the overall adjustment from Statsensor to lab was 0.69. Review of the Bland-Altman plots (Fig 1, Fig 2) both suggest slight biases at higher creatinine values, however it is much more apparent with the Statsensor.

**Table 1:** Comparison of mean point-of-care versus lab values among sugarcane workers in Guatemala, n = 89.

Biomarker	Lab	iSTAT	*p*-value	Statsensor*	*p*-value
	*Mean*, *SD*				
**Creatinine (mg/dL)**	0.80 (0.11)	0.80 (0.16)	0.91	1.12 (0.26)	<0.001
**Sodium (mmol/L)**	142 (2.05)	141 (1.79)	0.06	n/a	n/a
**Potassium (mmol/L)**	4.14 (0.38)	3.80 (0.44)	<0.001		
• Hypokalemia, < 3.5 mmol/L (n (%))	1 (1.1%)	18 (20.2%)			
Ionized calcium*	n/a	1.24 (0.04)	-		
**Anion gap (mmol/L)**	10.2 (1.67)	18.2 (1.37)	<0.001		
• High anion gap (potential acidosis), > 12 mmol/L (n(%))	7 (7.9%)	89 (100%)			
**TC02 (mmol/L)**	27.9 (2.05)	26.1 (2.10)	<0.001		
• Low TC02, < 20 mmol/L (n(%))	0 (0%)	1 (1.1%)			
**Hemoglobin (g/dL)**	14.8 (1.08)	15.3 (1.07)	0.001		
• Anemia, < 13 g/dL (n(%))	3 (3.4%)	3 (3.4%)			
**Glucose (mg/dL)**	82.8 (11.21)	97.7 (9.92)	<0.001		
• High glucose, > 100 mg/dL (n(%))	5 (5.6%)	22 (24.7%)			
**BUN (mg/dL)**	9.92 (2.27)	9.91 (3.69)	0.99		
**Hematocrit (%)**	41.1 (2.83)	45.1 (3.18)	<0.001		
• Low hematocrit, < 42% (n(%))	55 (61.8%)	9 (10.1%)			

*Only creatinine was measured by the Statsensor; ionized calcium was not measured by the lab.

Abbreviations: TC02: total carbon dioxide/bicarbonate; BUN: blood urea nitrogen

### Comparison of iSTAT and lab values by study day

**Fig 3:**
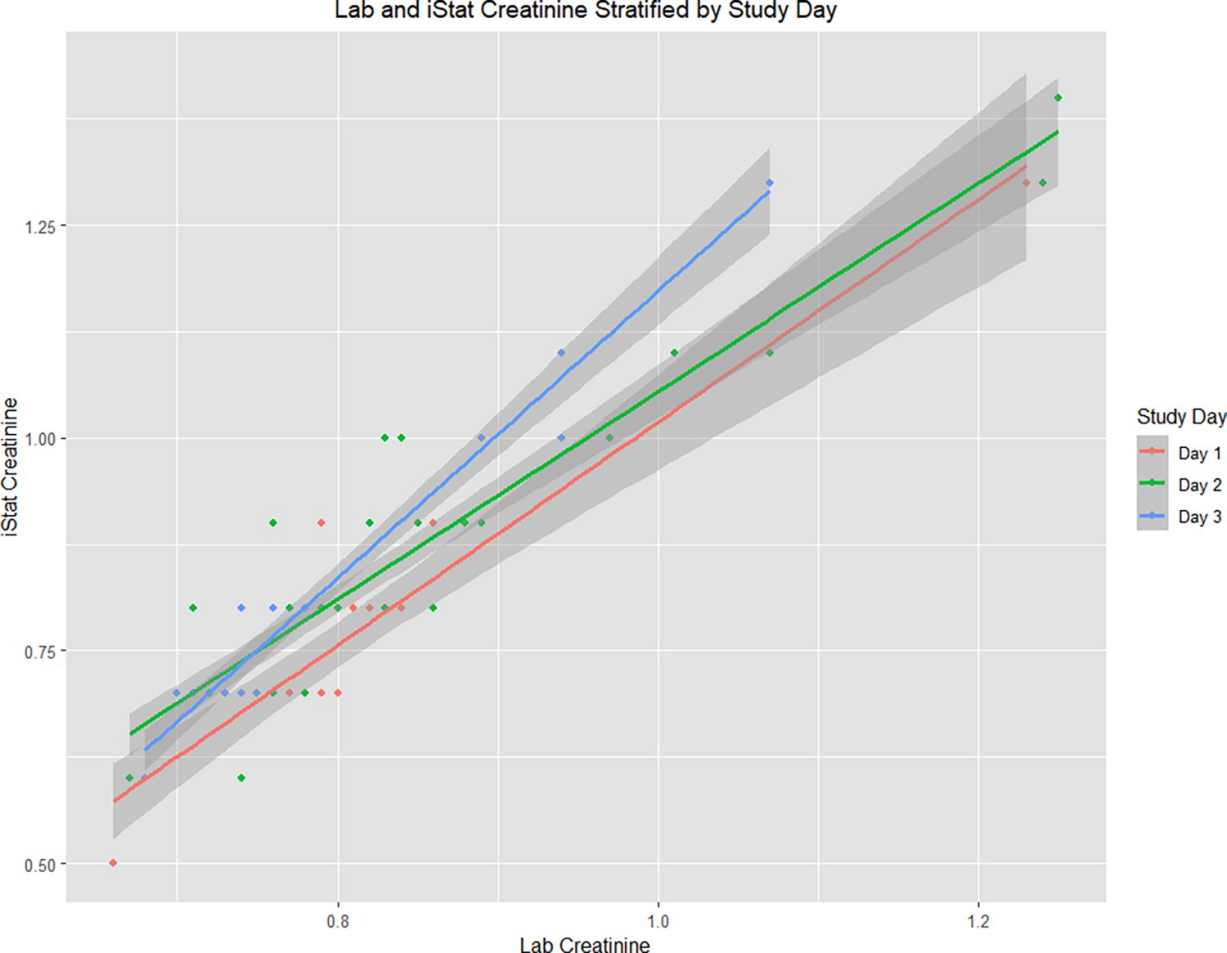
iSTAT and lab creatinine values, stratified by study day.

Table 2 shows the Pearson correlation coefficients and sample-specific correction factors between the iSTAT creatinine and corresponding lab measurements, stratified by day of data collection. As described above, overall, the iSTAT showed excellent correlation with the lab measure for creatinine, with slight differences in agreement between the iSTAT and lab creatinine depending on the study day (r = 0.93 on Day 1, 0.93 on Day 2, and 0.98 on Day 3, respectively). For creatinine, the iSTAT measures tended to underestimate the lab value on Day 1 whereas the iSTAT measures slightly overestimated the lab creatinine measures on Days 2 and 3 (Fig 3). We assessed these differences by day for electrolytes as well but did not observe a trend.

**Table 2:** Correlation of iSTAT and lab creatinine values.

Date	Pearson correlation coefficient	Sample-specific correction factor
Day 1	0.93	1.04
Day 2	0.93	0.98
Day 3	0.98	0.95

### Comparison of iSTAT and lab measurements

Table 1 displays the remaining iSTAT measurements compared with the concurrent lab values. We observed a trend with the electrolytes, as lab and iSTAT disagreed more at extreme electrolyte levels. The lab measurements tended to be higher than the iSTAT measures when electrolyte levels were higher, while the iSTAT values tended to be higher than lab values when electrolyte levels were lower. This tendency did not seem to impact measures of anion gap. The Bland-Altman plots of these relationships are included in the S1 Fig.

Overall, there was a strong linear association between lab sodium and iSTAT (142 mmol/L (SD: 2.05) versus 141 mmol/L (SD: 1.79), respectively)) *p* = 0.06. The BUN results were also not significantly different (lab: 9.92 mg/dL (SD: 2.27) versus 9.91 mg/dL (SD: 3.69), respectively (*p* = 0.99). However, the iSTAT tended to overestimate anion gap, hemoglobin, glucose, and hematocrit, and it underestimated potassium and TCO2, compared to the lab (*p* < 0.001).

In terms of clinical relevance of the differences observed between the lab and iSTAT, 20% of participants had low potassium (hypokalemia) using the iSTAT compared to 1% based on the lab measurement. For anion gap, the iSTAT placed all 89 participants (100%) above the normal range, indicating potential acidosis, compared to 8% based on the lab analysis. One worker (1%) had low TCO2 compared to none based on the lab measurments. Nearly 25% of participants had high glucose based on the iSTAT results, compared to 6% from the lab results. We observed the opposite trend with hematocrit, where 10% of participants had low hematocrit based on the iSTAT compared to 62% from the lab measurement. The prevalence of low hemoglobin (anemia) was the same (3%) for the two methods.

### Field application methods

**Fig 4:**
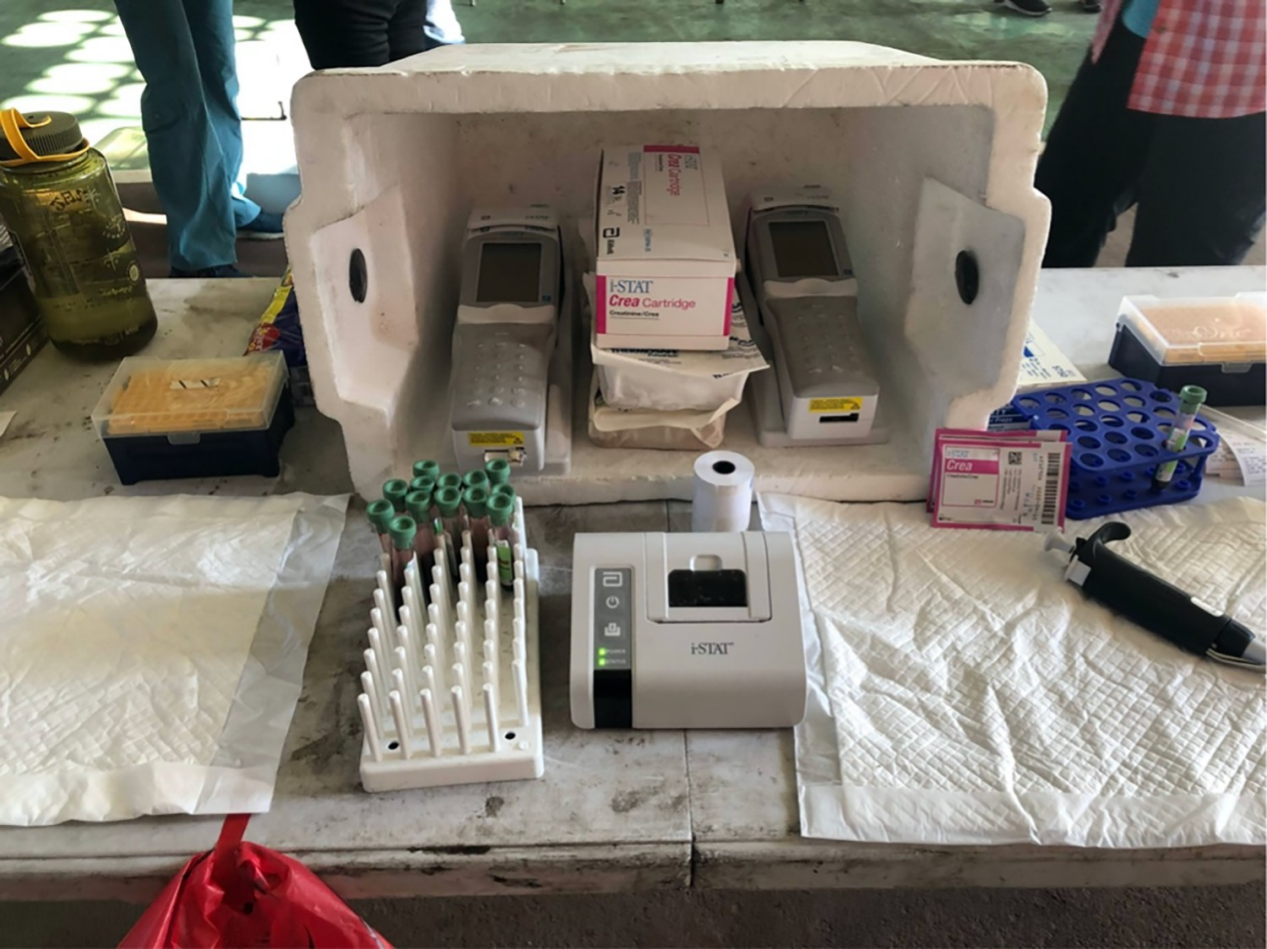
Picture of the iSTAT devices and test cartridges inside cooling shed with ice packs to prevent overheating in the field.

On Day 1 of data collection, the iSTAT meters both displayed a warning message several times that the temperature was out of range (operating range: 30-40°C), indicating that the analyzer was too warm. To address this, the field team constructed a cooling shed for the devices and cartridges using a Styrofoam cooler placed on its side on a table with several cold packs stacked inside along with the analysis cartridges (Fig 4). The team placed the iSTAT devices flat inside the open cooler where they remained during analysis. The devices were only removed to prepare for analysis (i.e., entering user and participant IDs, and scanning the cartridge barcode). The cartridges remained in the cooling shed until five minutes prior to analysis to reach room temperature (18–30°C), per the manufacturer’s instructions. These same methods were used in subsequent rounds of data collection, which took place directly in the sugarcane fields, under similar and often higher heat conditions during the 2021–2022 field campaign. Using these methods, iSTAT users did not see any error messages with the devices. The sampling campaigns lasted about 1.5 to two hours, both in the sugarcane fields and in the gymnasium where the current study was conducted.

We have observed that keeping the cooler open to the air, as depicted in Fig 4, is the optimal arrangement, since closing the cooler may result in the devices becoming too cold. Of note, on two recent occasions the team needed to shut the cooler during operation of the devices, due to extreme heat conditions in the sugarcane fields.

## Discussion

This study compared blood measures of underlying health status among Guatemalan sugarcane workers between two POC devices used in a tropical field setting, the iSTAT and Statsensor, with traditional methods from a locally accredited laboratory treated as the gold standard. The creatinine results showed better agreement with the lab using the iSTAT compared to the Statsensor, which showed bias especially at higher values of creatinine, consistent with previous studies [13, 17]. The iSTAT also showed good agreement with the lab for sodium and BUN, with some clinically relevant differences observed in the other biomarkers that were measured, including for potassium, anion gap, TCO2, glucose, and hematocrit, highlighting the importance of confirmatory lab testing for diagnostic and treatment purposes when using POC devices in the field. Our data indicate that handheld, battery-operated POC devices can be used successfully in tropical field settings, including those in which temperatures exceed the manufacturers’ claims for effective operating range (16-30°C in the case of the iSTAT, and 15–40°C for the Statsensor). These possible temperature limitations can be addressed using an operational protocol that includes the use of a cooler and ice packs to minimize heat exposure and keep these POC devices within prescribed operating ranges during transport and use in the field.

Studies that compare and validate POC devices with ‘gold-standard’ laboratory analysis can be found in the literature, with varying outcomes depending on the device and use setting. These comparisons can be difficult to make when it is not clear which method is most accurate. For example, it has been shown that the Jaffe method experiences positive bias in creatinine when there is delay in serum separation, which is a common circumstance in LMIC and especially rural areas [16, 23]. The Jaffe method is most common in LMIC in part because it is less expensive than the enzymatic method used elsewhere, however it is prone to interfering substances, including proteins, glucose and ketones that affect specificity [16, 24, 25]. Furthermore, with the increasing global prevalence of chronic kidney disease (CKD) of both traditional and non-traditional (CKDnT) causes [26], POC devices provide a useful and often more accessible solution for employers, healthcare systems, researchers, and communities, requiring less blood sample and quicker reporting of results. There is also value with POC devices in being able to provide immediate results with explanation to research participants and community members being screened. Some individuals may never have been tested or been provided with the results of their laboratory tests previously, providing a benefit to research and surveillance participation. The correction factors for creatinine, a measure of kidney function, that we observed in this study were consistent with those found in other published work in this area [15, 17].

In the present study comparing the creatinine results between the iSTAT and the Statsensor, the iSTAT showed greater accuracy compared to the Statsensor, as we have seen in our previous work [17]. This is especially important in situations that call for classification of CKD stage, or in employment medical exams that require a certain kidney function threshold for hiring, for example. For routine surveillance of kidney health, such as in high-risk communities and workplaces, the Statsensor, which is priced significantly lower than the iSTAT, requires only a finger prick blood sample, and is easier to use, may be satisfactory, with the caveat that the use of this device may report more false positives that will need to be verified with a second test [17]. A more detailed comparison between the iSTAT and Statsensor, including sensitivity and specificity statistics and the practical pros and cons of each device can be found in Dally et al., 2023 [17].

In terms of the other biomarkers that were compared between the iSTAT and lab, we observed some differences that would be considered clinically meaningful, highlighting that the iSTAT is not a perfect substitute for clinical lab measurement, particularly if the results are intended to be used for clinical decision making and treatment purposes. While there was a strong linear association between the lab and iSTAT for sodium and BUN, the iSTAT tended to overestimate the measurement for anion gap, hemoglobin, glucose, and hematocrit, while compared to the lab it underestimated potassium and TCO2. Although the difference in hemoglobin concentration between iSTAT and lab was statistically significant, we note that only 3% of individuals were below the normal range using either method, suggesting little clinical significance. On the other hand, the iSTAT placed 25% of individuals in the range of elevated glucose, compared to just 6% based on the lab result. For potassium, 20% would be considered hypokalemic [low potassium] based on the iSTAT, compared to just 1% using the lab measurement. This is useful because the iSTAT is used frequently for screening purposes, in which case some false positive results are acceptable in order to not miss potentially clinically important results, and with the assumption that the recommendation will be to obtain a clinical lab confirmation. However, it is important to note that the “risk” associated with false positives is that they may result in unnecessary worry on the part of the patient or participant and could increase expense for the individual and the health system created by the need to perform lab based tests and receive clinical evaluations for false positives. Importantly for hematocrit, the iSTAT produced more false negatives, placing just 10% of participants in the category of low hematocrit. The lab analysis resulted in 62% with low hematocrit, which is similar to what we observed in a previous study in a sugarcane worker population using the same laboratory in Guatemala [27]. This difference between the two methods warrants further investigation.

Finally, for creatinine, a possible explanation for the observed variation between the Statsensor and iSTAT may be attributable to differences between venous (iSTAT) and capillary (Statsensor) measurement (although the iSTAT can also be used with capillary samples). Hydration status is more likely to affect capillary measurements, which is an important consideration in remote field settings such as agricultural workplaces and communities where these devices are frequently used [13, 17]. This may explain why in a previous study among agricultural workers in Guatemala we found that a correction factor was necessary for the Statsensor in the afternoon, post-work shift, when workers were more likely to be dehydrated, but was found to not be necessary in pre-shift testing [15, 17]. We observed slight differences in agreement between the iSTAT and lab creatinine based on study day–for example, the iSTAT tended to underestimate creatinine on Day 1, which was also the hottest day based on the WBGT. Future research should examine this trend further to better understand the effect of environmental temperature on test results derived from POC devices.

A key strength of this study is that samples were collected for the laboratory analyses and the two POC devices concurrently, from the same individuals, allowing for direct comparison between the devices. In this study, as is customary, the laboratory measurements were treated as the gold standard, even though they may still be subject to bias as well. While the Jaffe method is commonly used in limited resource settings, the ability to compare these results to other laboratories using a different assay type or calibration standard as reference standard may be limited. Our sampling campaigns, both in the gymnasium where this study was conducted and subsequently in the sugarcane fields, lasted approximately 1.5 to two hours on average. We are unable to concude from this study whether the field application methods described here would be sufficient for a longer duration campaign in the heat.

This study provides further evidence of the utility of POC devices in LMIC and other low-resource field settings. These findings suggest that researchers and clinicians can use these devices to conduct high quality disease surveillance and provide timely results back to patients, study participants and communities.

## Supporting information

S1 Fig Bland-Altman plots of iSTAT measures with corresponding lab values. (DOCX)

S1 Table Comparison of reference ranges, point-of-care versus lab. (DOCX)

S1 Data Clinical data. (CSV)

S2 Data Weather data. (CSV)
