# Supplementary figures and images for: Application and comparison of point-of-care devices for field evaluation of underlying health status of Guatemalan sugarcane workers
Source: PLOS Glob Public Health. 2024 Jul 23;4(7):e0003380. doi: 10.1371/journal.pgph.0003380 (PMC11265697; doi:10.1371/journal.pgph.0003380)

| 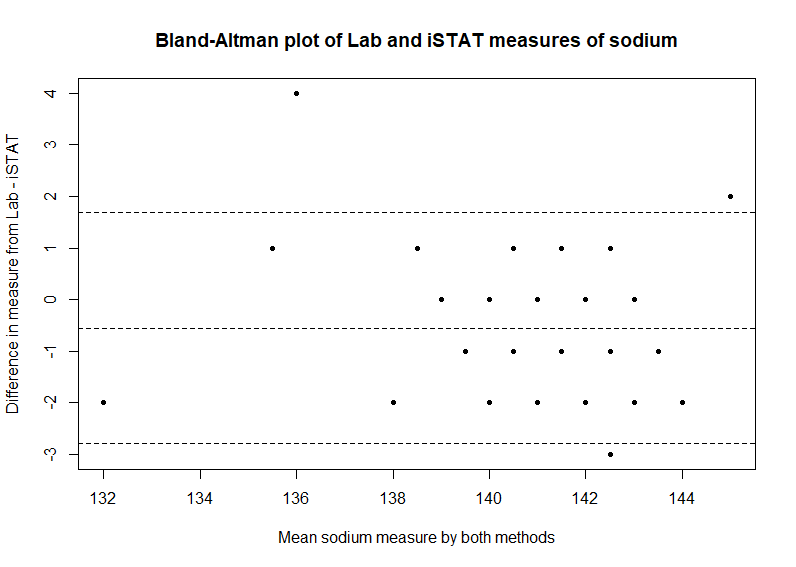 | 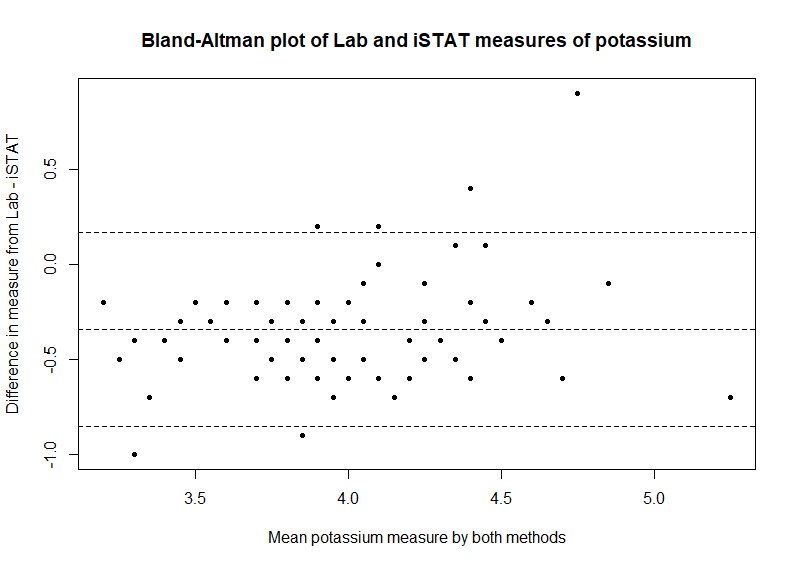 |
| --- | --- |
|  |  |
| 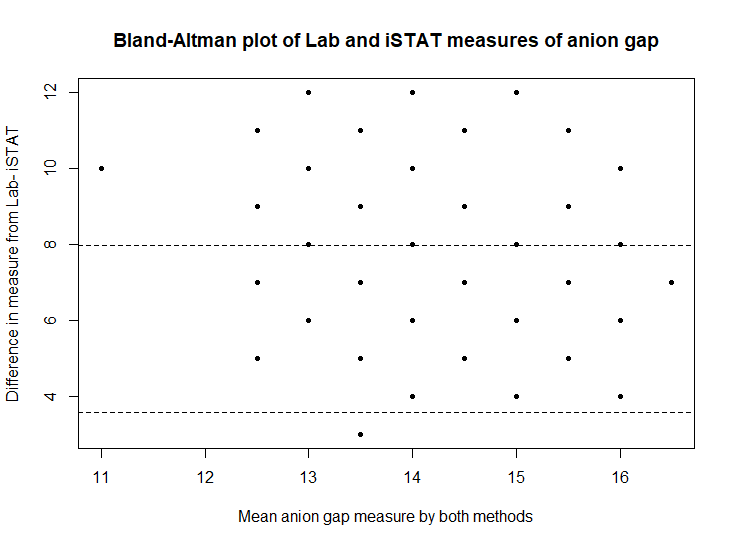 | 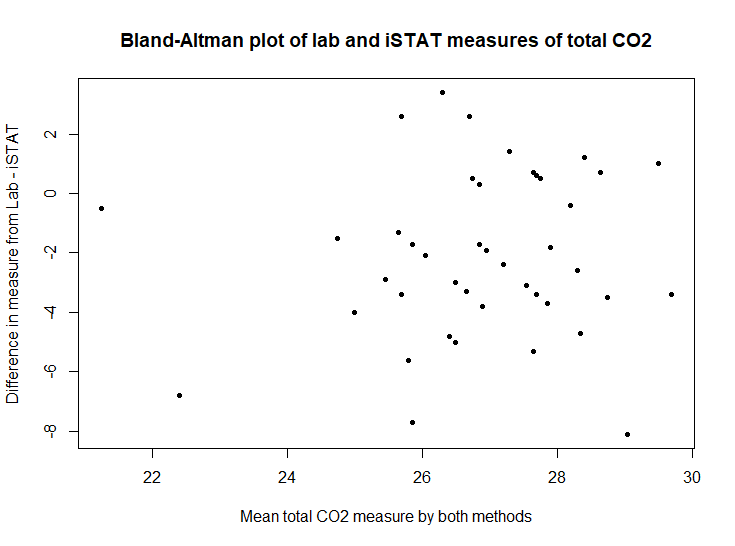 |
| 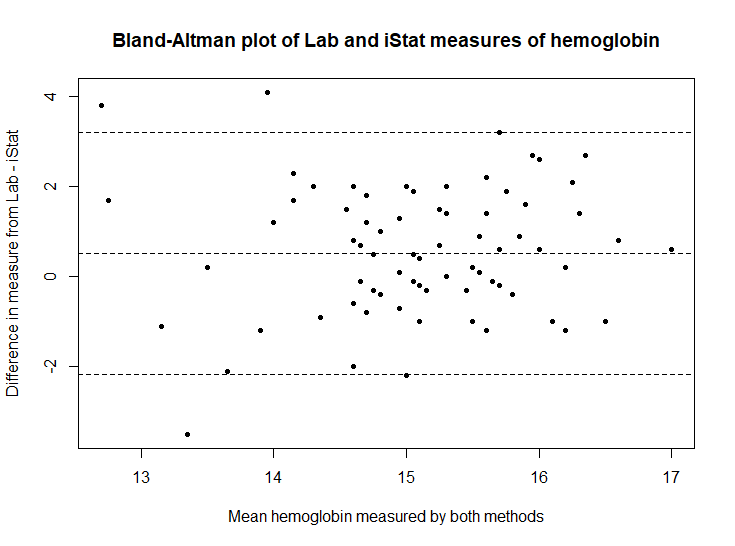 | 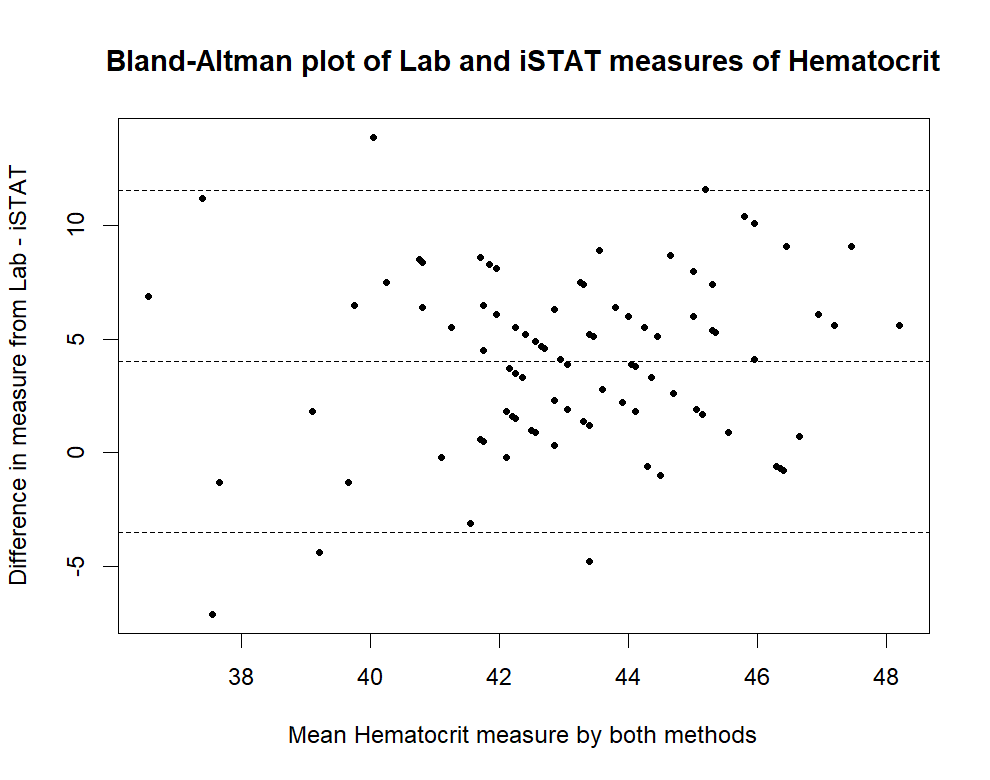 |
| 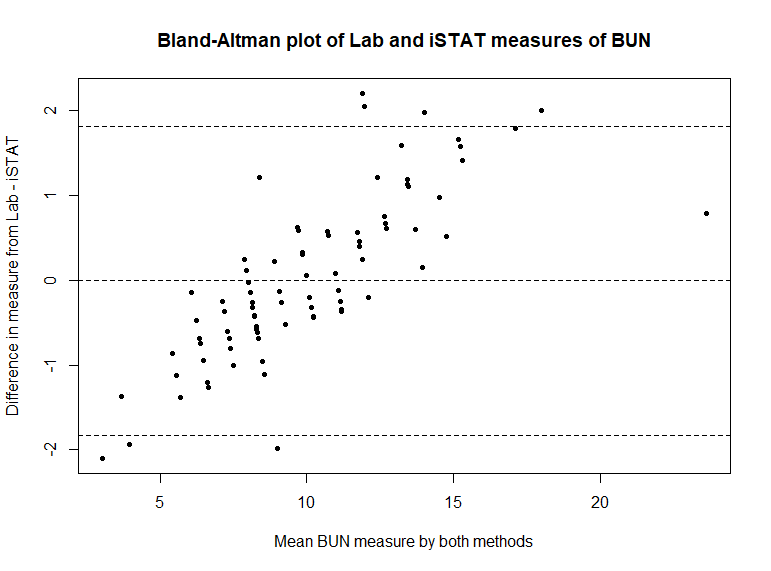 | 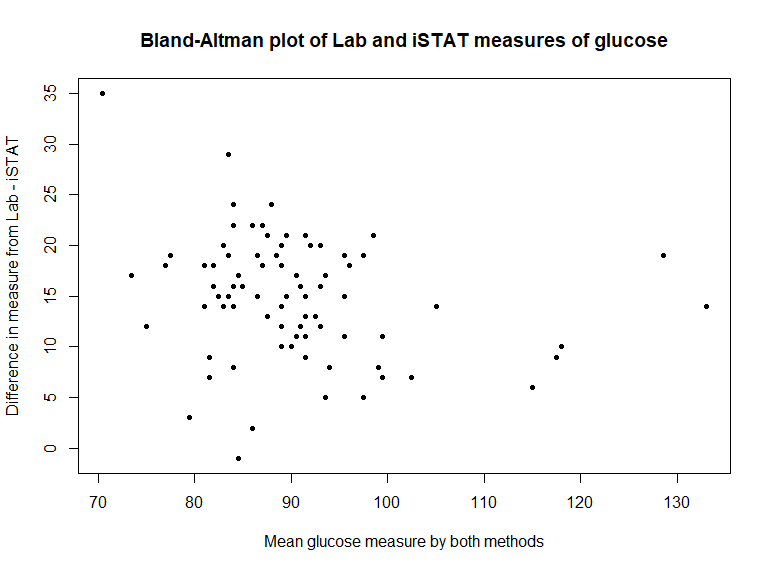 |

S1 Fig. Bland-Altman plots of iSTAT measures with corresponding lab values.

Supplement: S1 Fig — (DOCX) [file pgph.0003380.s001.docx]
