# Supplementary material for: Application and comparison of point-of-care devices for field evaluation of underlying health status of Guatemalan sugarcane workers
Source: PLOS Glob Public Health. 2024 Jul 23;4(7):e0003380. doi: 10.1371/journal.pgph.0003380 (PMC11265697; doi:10.1371/journal.pgph.0003380)
Supplement: S1 Table — (DOCX) [file pgph.0003380.s002.docx]

**S1 Table: Comparison of reference ranges, point-of-care versus lab**

| **Analyte** | **Laboratory Reference Range** | **iSTAT Reference Range** | **Statsensor*** |
| --- | --- | --- | --- |
| Creatinine (Crea) | 0.72-1.25 mg/dL | 0.60-1.30 mg/dL | 0.60-1.20 mg/dL |
| Sodium (Na) | 135-145 mmol/L | 138-146 mmol/L | n/a |
| Potassium (K) | 3.5-5.0 mmol/L | 3.5-4.9 mmol/L |  |
| Chloride (Cl)* | n/a | 98-109 mmol/L |  |
| TCO2 | 23-29 mmol/L | 24-29 mmol/L |  |
| Anion Gap | 10-20 mmol/L | 10-20 mmol/L |  |
| Ionized Calcium (iCa)* | n/a | 1.12-1.32 mmol/L |  |
| Glucose (Glu) | 70-110 mg/dL | 70-105 mg/dL |  |
| Urea Nitrogen (BUN)/Urea | 8.9-20.6 mg/dL | 8-26 mg/dL |  |
| Hematocrit (Hct) | 42-50 % | 38-51 % |  |
| Hemoglobin (Hgb) | 13-18 g/dL | 12-17 g/dL |  |

**Only creatinine was measured by the Statsensor; ionized calcium and chloride were not measured by the lab. Abbreviations: TC02: total carbon dioxide/bicarbonate;*
